# Risk factors for reoperation of inflatable penile prosthesis among an ethnically diverse urban population in a high-volume center

**DOI:** 10.1038/s41443-024-00966-8

**Published:** 2024-08-26

**Authors:** Noah Hawks-Ladds, Mustufa Babar, Kevin Labagnara, Justin Loloi, Rutul D. Patel, Arshia Aalami Harandi, Michael Zhu, Azizou Salami, Pedro Maria

**Affiliations:** 1 Albert Einstein College of Medicine, Bronx, NY USA; 2 Department of Internal Medicine, Donald and Barbara Zucker School of Medicine at Hofstra/Northwell, Long Island, NY USA; 3 Department of Urology, Montefiore Medical Center, Bronx, NY USA; 4 Department of Urology, Stony Brook University Hospital, Stony Brook, NY USA

**Keywords:** Erectile dysfunction, Quality of life, Surgery

## Abstract

Inflatable penile prosthesis (IPP) is a surgical treatment for erectile dysfunction refractory to medical therapy or for those who desire permanent treatment. Complications like mechanical failure and infection may necessitate reoperation, and patients with certain risk factors remain predisposed to reoperation. We retrospectively analyzed 530 patients undergoing primary IPP implantation at a large, urban, multiethnic hospital with a high volume of IPP implantations. Primary outcomes were reoperation due to any reason and reoperation due to infection. Patient characteristics and intraoperative factors were compared between those requiring reoperation and those not requiring reoperation. Overall, 12.1% of patients underwent reoperation, primarily due to infection, with a median time to reoperation of 4 months. Analysis revealed an increased likelihood of reoperation with Peyronie’s disease (OR = 2.47), hemoglobin A1c over 8 (OR = 2.25), active smoking (OR = 2.75), and estimated blood loss (EBL) ≥ 25cc (OR = 2.45). A decreased likelihood of reoperation was observed when Arista™ powder was used intraoperatively (OR = 0.38). Reoperation specifically due to infection was associated with an infrapubic approach (OR = 2.56) and hypertension (OR = 9.12). Our findings confirm smoking and diabetes as risk factors for reoperation, while also providing insights into factors like estimated blood loss and Arista™ powder use. However, long-term survival rates were limited by loss to follow-up. (Clinical trial registration N/A).

## Introduction

Erectile dysfunction (ED), defined as the inability to achieve or maintain an erection adequate for satisfactory sexual intercourse, increases in prevalence with age, affecting up to 37% of men between 70 and 75 years old [1–3]. Pharmacologic therapy for ED may induce adverse side effects in some patients and up to 30% of ED patients may demonstrate failure of medical treatment [4, 5].

Inflatable penile prosthesis (IPP) implantation stands as a popular surgical treatment option for ED. Historically, IPP implantation had been reserved for those who fail medical therapy, however, more recent literature suggests that it may be the best initial treatment option depending on the clinical scenario, for example in those who desire permanent treatment [6, 7]. IPP surgery consistently yields high rates of patient satisfaction, reported as high as 92% in some studies [8, 9]. Nonetheless, postoperative complications, such as mechanical failure, cylinder erosion, and infection, necessitate reoperation [10]. Among the common postoperative complications, prosthesis infection has garnered significant attention. Advancements in sterile technique, perioperative antibiotics, and infection retardant coatings have greatly improved infection prevention [11–13]. Despite these measures, many patients remain at risk for postoperative complications and subsequent reoperation. Risk factors such as diabetes mellitus, secondary implantation, surgeon inexperience, smoking, and concomitant procedures have been identified as contributors of reoperation [14–16].

However, there remains a paucity of data exploring these risk factors for reoperation after IPP implantation, particularly in a diverse, multiethnic patient population. This is particularly pertinent as African-American and Hispanic men seemingly face the highest risk for reoperation [17]. Thus, to address this gap in the literature, we investigate risk factors within a multiethnic, urban patient population at a high-volume center with high comorbidity rates to analyze the risk of reoperation after IPP surgery, with specific focus on those patients requiring reoperation due to prosthesis infection.

## Materials and methods

This study was a retrospective review of patients receiving primary IPP implantation surgery at our large, multiethnic, urban hospital. Institutional review board approval (IRB No. 2021-13240) was obtained prior to study commencement. The data collection period spanned from January 2015, when the highest-volume IPP surgeon at the institution started practicing, to December 2022, when the sample size was deemed to provide adequate statistical power. Patients undergoing semi-rigid prothesis placement and those with a prior IPP-related surgery were excluded from the study. The primary outcomes were reoperation due to any reason and reoperation specifically due to infection.

Electronic medical records prior to surgery were queried for demographic data such as age, race, preferred language, BMI, and medical comorbidities. Comorbidities collected include diabetes, hypertension, cardiac disease, hyperlipidemia, chronic kidney disease (CKD), pre-operative hemoglobin A1c levels, radical prostatectomy, radiation therapy, Peyronie’s disease, and sickle cell disease/priapism. Patients undergoing a concurrent procedure such as circumcision, penile modeling, and/or others were accounted for and included in the analysis. Operative logs were accessed for intra-operative outcome metrics such as surgical duration, drain usage, and estimated blood loss (EBL). IPP device specifications such as cylinder size (cm), reservoir size (cc), and rear tip extender use were recorded from operative notes, along with whether Arista™ AH absorbable hemostatic agent (Arista™; Davol Inc., Warwick, RI) was applied. 30-day safety secondary outcomes recorded included visits to the Emergency Department.

Patient characteristics, implant characteristics, intraoperative factors, and 30-day outcomes were collected. Percentages and proportions were calculated based on the subset of patients with available data for each specific variable, rather than the total sample size. Outcomes were compared between those who needed a reoperation (yes/no) via Chi-square test for categorical variables, student’s *t* test for continuous variables, and Mann-Whitney U test for skewed continuous variables. Cox proportional hazard regression models were used for univariate and multivariate survival analysis of risk factors for reoperation, and subsequently for infection. Final multivariate models were adjusted for age, BMI, smoking status, and infrapubic surgical approach.

Kaplan-Meier survival analysis was used to depict the 5-year any-reason reoperation rates and 1-year reoperation rates due to infection. Implant survival was defined as the continued presence of the primary implant without reoperation or removal surgery, as documented at specified time intervals during in-person follow-up. All p-values are two-sided with statistical significance set at p < 0.05. All statistical analyses were performed using SPSS version 28.0 (IBM, Armonk, NY).

## Results

A total of 530 patients underwent a primary IPP implantation within the study period, with 64 patients (12.1%) requiring a reoperation (Table 1). The overall median (IQR) follow-up time of our population was 14 (4-34) months and the median (IQR) time to reoperation was 4 (2-16) months. 29 (45.3%) reoperations were due to infection, 14 (21.9%) due to device malfunction, and 21 (32.8%) due to less common causes such as trauma, wound dehiscence, loss of dexterity, and esthetic concerns. The median (IQR) age of our population was 64 (58-68) years, with over two-thirds being of Hispanic ethnicity (n = 365, 69.4%) and reporting Spanish as their preferred language (67.0%). Median BMI was similar between those who did not need a reoperation (28.3, IQR: 25.8-31.7) and those who did (29.2, IQR: 25.8-33.9) (p = 0.25). Although not significant, rates of Peyronie’s disease (10.9% vs 5.2%, p = 0.064), diabetes (57.3% vs 48.4%, p = 0.18), hypertension (78.1% vs 73.6%, p = 0.44), and cardiac disease (28.1% vs 22.7%, p = 0.34) were elevated in the reoperation group. The median (IQR) hemoglobin A1c was 7.4 [6.0–8.4] in the reoperation group, compared to 6.8 [5.8–8.0] in the no reoperation group (p = 0.19). The proportion of former (35.9% vs 28.1%) and active smokers (14.1% vs 6.7%) was significantly greater (p = 0.025) in the reoperation group, while no differences were noted in those with a history of hyperlipidemia, CKD, prostatectomy, or radiation therapy (all p > 0.05).

**Table 1:** Baseline patient characteristics, intra-operative outcomes, and post-operative outcomes of those who underwent a reoperation for any reason versus those who did not.

	All patients	No reoperation	Reoperation	p value
**N (%)**	530	466 (87.9)	64 (12.1)	
Age, median (IQR)	64 (58–68)	64 (58–68)	63 (58–69)	0.79
BMI, median (IQR)	28.4 (25.8-31.8)	28.3 (25.8-31.7)	29.2 (25.8-33.9)	0.25
Race, N (%)				0.73
Non-Hispanic White	10 (1.9)	8 (1.7)	2 (3.1)	
Non-Hispanic Black	73 (13.9)	62 (13.4)	11 (17.2)	
Hispanic	365 (69.4)	323 (69.9)	42 (65.6)	
Other	78 (14.8)	69 (14.9)	9 (14.1)	
Preferred Language, N (%)				0.41
English	171 (32.4)	148 (31.9)	23 (35.9)	
Spanish	354 (67.0)	314 (67.7)	40 (62.5)	
Other	3 (0.6)	2 (0.4)	1 (1.6)	
Comorbidities, N (%)				
Prostatectomy	124 (23.4)	112 (24.0)	12 (18.8)	0.35
XRT	46 (8.7)	41 (8.8)	5 (7.8)	0.79
Peyronie’s Disease	31 (5.8)	24 (5.2)	7 (10.9)	0.064
Sickle Cell/Priapism	6 (1.1)	3 (0.6)	3 (4.7)	**0.040**
Diabetes	298 (56.2)	267 (57.3)	31 (48.4)	0.18
Hemoglobin A1c, median (IQR)	6.8 (5.8–8.1)	6.8 (5.8–8.0)	7.4 (6.0–8.4)	0.19
Hypertension	393 (74.2)	343 (73.6)	50 (78.1)	0.44
Hyperlipidemia	267 (50.4)	237 (50.9)	30 (46.9)	0.55
Cardiac Disease	124 (23.4)	106 (22.7)	18 (28.1)	0.34
Chronic Kidney Disease	77 (14.5)	68 (14.6)	9 (14.1)	0.91
Smoker, N (%)				**0.025**
Never	336 (63.4)	304 (65.2)	32 (50.0)	
Former	154 (29.1)	131 (28.1)	23 (35.9)	
Active	40 (7.5)	31 (6.7)	9 (14.1)	
Operative time (min), median (IQR)	65 (55–79)	64 (55–77)	68 (52–89)	0.31
Approach, N (%)				0.78
Penoscrotal	282 (53.2)	249 (53.4)	33 (51.6)	
Infrapubic	248 (46.8)	217 (46.6)	31 (48.4)	
Concurrent Surgery, N (%)				
Any	141 (26.6)	122 (26.2)	19 (29.7)	0.55
Circumcision	102 (19.2)	88 (18.9)	14 (21.9)	0.57
Penile Modeling	44 (8.3)	38 (8.2)	6 (9.4)	0.74
Other	5 (0.9)	4 (0.9)	1 (1.6)	0.59
Manufacturer, N (%)				0.17
Coloplast	499 (94.3)	441 (94.8)	58 (90.6)	
American Medical Systems	30 (5.7)	24 (5.2)	6 (9.4)	
Implant Cylinder Size (cm), N (%)				0.29
<18	55 (10.4)	44 (9.5)	11 (17.2)	
18–19	191 (36.1)	171 (36.8)	20 (31.3)	
20–21	192 (36.3)	170 (36.6)	22 (34.4)	
22+	91 (17.2)	80 (17.2)	11 (17.2)	
Reservoir Size ≥ 100cc, N (%)	191 (36.4)	170 (36.8)	21 (33.3)	0.59
Rear Tip Extender, N (%)	266 (50.4)	237 (51.1)	29 (45.3)	0.39
Surgical Drain, N (%)	27 (5.1)	21 (4.5)	6 (9.4)	0.097
EBL (cc), median (IQR)	25 (15-50)	25 (10–50)	30 (25-50)	**0.035**
Arista™ Powder Usage, N (%)	176 (33.2)	166 (35.6)	10 (15.6)	**<0.001**
Discharged with catheter, N (%)	45 (8.5)	39 (8.4)	6 (9.4)	0.79
30-Day ED Presentation, N (%)	65 (12.3)	54 (11.6)	11 (17.2)	0.20
Pain/Swelling	45 (71.4)	42 (77.8)	3 (33.3)	
Retention	10 (15.9)	5 (9.3)	5 (55.6)	
Other	8 (12.7)	7 (13.0)	1 (11.1)	

Non-normally distributed variables are reported as median (IQR).

Categorical variables are reported as N (%).

Characteristics were compared using Mann–Whitney U tests and Chi-squared analysis.

Bold values indicate statistical significance *p* < 0.05.

*IQR* interquartile range, *BMI* body mass index, *XRT* history of radiation therapy, *min* minutes, *cm* centimeters, *EBL* estimated blood loss, *cc* cubic centimeter, *ED* emergency department.

Median (IQR) operative time was 65 min (55–79) and slightly greater in those who needed a subsequent reoperation (68 [52–89] vs 64 [55–77], p = 0.31) (Table 1). 141 (26.6%) patients underwent a concurrent operation which consisted mostly of circumcisions (n = 102, 19.2%) and penile modeling (n = 44, 8.3%). 94.3% of IPPs inserted were manufactured by Coloplast (Coloplast Corp., Minneapolis, MN), and the majority utilized an implant size of either 18–19 cm (36.1%) or 20–21 cm (36.3%). Rear tip extenders were used in 49.3% of cases and a reservoir size ≥ 100cc was used in 36.5% of cases. Median EBL in the reoperation group was 30cc [IQR: 25-50], compared to 25cc [10–50] in the no reoperation cohort (p = 0.035). Arista™ powder was used in a significantly smaller proportion of reoperation cases (15.6% vs 35.6%, p = 0.001). Although not significant, rates of surgical drain insertion were greater in the reoperation cohort (9.4% vs 4.5%, p = 0.095). Post-operative catheter rates were similar between groups (p = 0.79), although rates of 30-day ED presentation were slightly greater in the reoperation group (17.2% vs 11.6%, p = 0.20) and were mostly for urinary retention (55.6% vs 9.3%).

Demographic, intra-operative, and post-operative variables were then compared for those who needed a reoperation due to IPP infection (Table 2). Statistically significant differences were noted in the median (IQR) level of hemoglobin A1c (7.4 [6.0–8.4] vs 6.8 [5.8–8.0], p = 0.039), hypertension rates (96.6% vs 72.9%, p = 0.005), infrapubic surgical approach (65.5% vs 45.7%, p = 0.038), and 30-day ED presentation (24.1% vs 11.6%, p = 0.045) that were not present previously. The difference in smoking rates widened for both former smokers (37.9% vs 28.5%) and active smokers (20.7% vs 6.8%, p = 0.006).

**Table 2:** Baseline characteristics, intra-operative outcomes, and post-operative outcomes of those who had a reoperation due to infection versus those who did not.

	All Patients	No Infection	Infection	p value
**N (%)**	530	501 (94.5)	29 (5.5)	
Age, median (IQR)	64 (58–68)	64 (59–68)	62 (58–67)	0.41
BMI, median (IQR)	28.4 (25.8–31.8)	28.3 (25.8–31.7)	29.1 (25.8–34.3)	0.48
Race, N (%)				0.25
Non-Hispanic White	10 (1.9)	9 (1.8)	1 (3.4)	
Non-Hispanic Black	73 (13.9)	66 (13.3)	7 (24.1)	
Hispanic	365 (69.4)	346 (69.6)	19 (65.5)	
Other	78 (14.8)	76 (15.3)	2 (6.9)	
Preferred Language, N (%)				0.099
English	171 (32.4)	161 (32.3)	10 (34.5)	
Spanish	354 (67.0)	336 (67.3)	18 (62.1)	
Other	3 (0.6)	2 (0.4)	1 (3.4)	
Comorbidities, N (%)				
Prostatectomy	124 (23.4)	120 (20.4)	4 (13.8)	0.21
XRT	46 (8.7)	43 (8.6)	3 (10.3)	0.74
Peyronie’s Disease	31 (5.8)	27 (5.4)	4 (13.8)	0.061
Sickle Cell/Priapism	6 (1.1)	5 (1.0)	1 (3.4)	0.23
Diabetes	298 (56.2)	281 (56.1)	17 (58.6)	0.79
Hemoglobin A1c, median (IQR)	6.8 (5.8–8.1)	6.8 (5.8–8.0)	8.1 (6.8–8.5)	**0.039**
Hypertension	393 (74.2)	365 (72.9)	28 (96.6)	**0.005**
Hyperlipidemia	267 (50.4)	253 (50.5)	14 (48.3)	0.82
Cardiac Disease	124 (23.4)	116 (23.2)	8 (27.6)	0.58
Chronic Kidney Disease	77 (14.5)	72 (14.4)	5 (17.2)	0.67
Smoker, N (%)				**0.006**
Never	336 (63.4)	324 (64.7)	12 (41.4)	
Former	154 (29.1)	143 (28.5)	11 (37.9)	
Active	40 (7.5)	34 (6.8)	6 (20.7)	
Operative time (min), median (IQR)	65 (55–79)	64 (55–78)	76 (55–86)	0.18
Approach, N (%)				**0.038**
Penoscrotal	282 (53.2)	272 (54.3)	10 (34.5)	
Infrapubic	248 (46.8)	229 (45.7)	19 (65.5)	
Concurrent Surgery, N (%)				
Any	141 (26.6)	131 (26.1)	10 (34.5)	0.32
Circumcision	102 (19.2)	94 (18.8)	8 (27.6)	0.24
Penile Modeling	44 (8.3)	41 (8.2)	3 (10.3)	0.068
Other	5 (0.9)	4 (0.8)	1 (3.4)	0.15
Manufacturer, N (%)				0.59
American Medical Systems	30 (5.7)	29 (5.8)	1 (3.4)	
Coloplast	499 (94.3)	471 (94.2)	28 (96.6)	
Implant Cylinder Size (cm), N (%)				0.57
<18	55 (10.4)	50 (10.0)	5 (17.2)	
18–19	191 (36.1)	183 (36.6)	8 (27.6)	
20–21	192 (36.3)	181 (36.2)	11 (37.9)	
22+	91 (17.2)	86 (17.2)	5 (17.2)	
Reservoir Size ≥ 100cc, N (%)	191 (36.4)	182 (36.7)	9 (31.0)	0.54
Rear Tip Extender, N (%)	266 (50.4)	253 (50.7)	13 (44.8)	0.54
Surgical Drain, N (%)	27 (5.1)	25 (5.0)	2 (6.9)	0.65
EBL (cc), median (IQR)	25 (15-50)	25 (14-50)	30 (23-50)	0.29
Arista™ Powder Usage, N (%)	176 (33.2)	169 (33.7)	7 (24.1)	0.29
Discharged with catheter, N (%)	45 (8.5)	43 (8.6)	2 (6.9)	0.75
30-Day ED Presentation, N (%)	65 (12.3)	58 (11.6)	7 (24.1)	**0.045**
Pain/Swelling	45 (71.4)	43 (75.4)	2 (33.3)	
Retention	10 (15.9)	7 (12.3)	3 (50.0)	
Other	8 (12.7)	7 (12.3)	1 (16.7)	

Non-normally distributed variables are reported as median (IQR).

Categorical variables are reported as N (%).

Characteristics were compared using Mann–Whitney U tests and Chi-squared analysis.

Bold values indicate statistical significance *p* < 0.05.

*IQR* interquartile range, *BMI* body mass index, *XRT* history of radiation therapy, *min* minutes, *cm* centimeters, *EBL* estimated blood loss, *cc* cubic centimeter, *ED* emergency department.

**Fig. 1:**
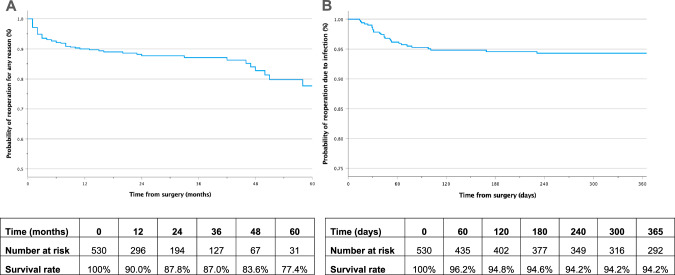
Kaplan Meier curves estimating implant survival. **A** 5-year reoperation survival rates for any reason. **B** 1-year reoperation survival rates due to infection. The tables below each curve show the number of subjects at risk at significant time intervals.

Kaplan Meier survival curves were generated to visualize the 5-year reoperation rates due to any reason (Figs. 1A) and 1-year reoperation rates due to infection (Fig. 1B). The reoperation survival rate for any reason at 1 year was 90%, declining to 77.4% by 5 years. The reoperation survival rate due to infection was 96.2% within 60 days, decreasing to 94.2% within 1 year.

Cox proportional hazard models were then utilized to analyze the association of demographic and intra-operative risk factors to reoperation in general and due to infection. (Table 3). After adjusting for the potential confounding effects of age, BMI, smoking status, and surgical approach, multivariate analysis revealed a significantly elevated likelihood for any-time reoperation with Peyronie’s disease (OR = 2.47, 95% CI: 1.10-5.58, p = 0.029), hemoglobin A1c ≥ 8.0 (OR = 2.25, 95% CI: 1.07-4.72, p = 0.029), active smokers (OR = 2.75, 95% CI: 1.30-5.85, p = 0.008), and EBL ≥ 25cc (OR = 2.45, 95% CI: 1.16-5.19, p = 0.019), while a decreased likelihood was observed when Arista™ powder was used intraoperatively (OR = 0.38, 95% CI: 0.18-0.81, p = 0.012). When analyzing only reoperations due to infection, factors that remained significant on multivariate analysis include hemoglobin A1c ≥ 8.0 (p = 0.017), actively smoking (p = 0.008), and Arista™ powder usage (p = 0.047). Utilizing an infrapubic approach (OR = 2.56, 95% CI: 1.15-5.68, p = 0.021) and a pre-operative diagnosis of hypertension (OR = 9.12, 95% CI = 1.23-67.59, p = 0.031) were significantly associated with cases of infection. Although only significant at one size for reoperations in general, larger cylinder sizes overall had lower odds of reoperation.

**Table 3:** Univariate and multivariate survival analysis for reoperation risk factors using cox proportional hazard models.

	Reoperation				Infection			
Characteristics	Univariate		Multivariate		Univariate		Multivariate	
Pre-operative	OR (95% CI)	P value	OR (95% CI)	P value	OR (95% CI)	P value	OR (95% CI)	P value
Age	1.01 (0.98–1.04)	0.74	1.01 (0.98–1.04)	0.63	0.99 (0.95–1.03)	0.64	0.99 (0.95–1.04)	0.70
BMI	1.03 (0.98–1.08)	0.31	1.02 (0.97–1.07)	0.49	1.03 (0.96–1.11)	0.43	1.01 (0.94–1.09)	0.79
Race (ref = Hispanic)								
Non-Hispanic White	2.12 (0.51–8.80)	0.30	1.73 (0.41–7.37)	0.46	2.15 (0.29–16.09)	0.46	1.89 (0.25–14.33)	0.54
Non-Hispanic Black	1.24 (0.64–2.42)	0.52	1.11 (0.56–2.20)	0.76	1.82 (0.77–4.33)	0.18	1.53 (0.63–3.71)	0.35
Other	1.04 (0.51–2.16)	0.91	1.10 (0.53–2.28)	0.80	0.53 (0.12–2.28)	0.39	0.57 (0.13–2.45)	0.45
Comorbidities								
Prostatectomy	0.68 (0.36–1.27)	0.22	0.74 (0.39–1.40)	0.35	0.50 (0.17–1.44)	0.20	0.61 (0.21–1.78)	0.36
XRT	0.83 (0.33–2.07)	0.69	0.75 (0.30–1.90)	0.55	1.18 (0.36–3.90)	0.79	1.04 (0.31–3.48)	0.95
Peyronie’s Disease	2.52 (1.15–5.54)	**0.022**	2.47 (1.10–5.58)	**0.029**	2.92 (1.01–8.43)	**0.047**	2.45 (0.82–7.35)	0.11
Diabetes	0.76 (0.47–1.24)	0.28	0.72 (0.44–1.19)	0.21	1.10 (0.53–2.30)	0.80	1.11 (0.52–2.36)	0.78
Hemoglobin A1c ≥ 8.0	2.22 (1.09–4.52)	**0.029**	2.25 (1.07–4.72)	**0.032**	3.13 (1.21–8.13)	**0.019**	3.29 (1.24–8.76)	**0.017**
Hypertension	1.13 (0.63–2.06)	0.68	1.03 (0.56–1.88)	0.93	9.58 (1.30–70.47)	**0.026**	9.12 (1.23–67.59)	**0.031**
Cardiac Disease	1.31 (0.76–2.26)	0.34	1.18 (0.67–2.05)	0.57	1.25 (0.55–2.82)	0.60	1.10 (0.48–2.51)	0.83
Hyperlipidemia	0.96 (0.59–1.57)	0.86	0.88 (0.53–1.46)	0.62	0.97 (0.47–2.01)	0.93	0.86 (0.41–1.83)	0.70
Chronic Kidney Disease	0.90 (0.44–1.81)	0.76	0.79 (0.39–1.62)	0.52	1.15 (0.44–3.01)	0.78	1.03 (0.39–2.75)	0.95
Smoker (ref = Never)								
Former	1.54 (0.89–2.64)	0.12	1.56 (0.90–2.69)	0.11	1.94 (0.85–4.40)	0.11	2.04 (0.89–4.67)	0.090
Active	2.55 (1.20–5.38)	**0.014**	2.75 (1.30–5.85)	**0.008**	4.13 (1.54–11.11)	**0.005**	4.61 (1.71–12.42)	**0.002**
**Intra-operative**								
Operative Time	1.01 (0.99–1.02)	0.13	1.01 (0.99–1.02)	0.30	1.01 (0.99–1.03)	0.13	1.01 (0.99–1.02)	0.43
Infrapubic Approach	1.46 (0.88–2.43)	0.14	1.52 (0.89–2.58)	0.13	2.38 (1.10–5.14)	**0.027**	2.56 (1.15–5.68)	**0.021**
Concurrent Surgery								
Any	1.30 (0.76–2.23)	0.35	1.39 (0.80–2.39)	0.24	1.57 (0.73–3.41)	0.25	1.79 (0.82–3.90)	0.14
Circumcision	1.20 (0.66–2.18)	0.55	1.34 (0.73–2.46)	0.34	1.64 (0.72–3.72)	0.24	2.31 (0.98–5.43)	0.055
Penile Modeling	1.43 (0.61–3.32)	0.41	1.35 (0.57–3.22)	0.50	1.43 (0.43–4.74)	0.56	1.11 (0.32–3.85)	0.87
Cylinder Size (ref<18cm)								
18–19	0.50 (0.24–1.05)	0.068	0.47 (0.22–0.99)	**0.045**	0.45 (0.15–1.39)	0.17	0.45 (0.14–1.40)	0.17
20–21	0.62 (0.30–1.29)	0.20	0.63 (0.30–1.32)	0.22	0.64 (0.22–1.86)	0.41	0.64 (0.22–1.89)	0.42
22+	0.71 (0.30–1.67)	0.43	0.61 (0.26–1.44)	0.26	0.62 (0.18–2.18)	0.46	0.50 (0.14–1.78)	0.28
Reservoir Size ≥ 100cc	0.85 (0.50–1.44)	0.55	0.86 (0.50–1.47)	0.58	0.78 (0.36–1.72)	0.54	0.86 (0.39–1.94)	0.72
Rear Tip Extender	0.83 (0.51–1.36)	0.46	0.75 (0.45–1.24)	0.26	0.80 (0.39–1.67)	0.56	0.70 (0.33–1.47)	0.34
Surgical Drain	1.86 (0.80–4.32)	0.15	1.91 (0.81–4.47)	0.14	1.40 (0.33–5.88)	0.65	1.25 (0.29–5.35)	0.77
EBL ≥ 25cc	2.33 (1.11–4.89)	**0.026**	2.45 (1.16–5.19)	**0.019**	2.36 (0.77–7.25)	0.13	2.26 (0.73–6.99)	0.16
Arista™ Powder Usage	0.55 (0.27–1.10)	0.090	0.38 (0.18–0.81)	**0.012**	0.69 (0.29–1.63)	0.40	0.39 (0.16–0.99)	**0.047**

Due to high number of missing variables, multivariate model is only adjusted using the continuous forms of age and BMI, along with smoking status and infrapubic approach.

Bold values indicate statistical significance *p* < 0.05.

*OR* odds ratio, *CI* confidence interval, *BMI* body mass index, *ref* reference, *XRT* history of radiation therapy, *cm* centimeters, *cc* cubic centimeter, *EBL* estimated blood loss.

## Discussion

IPPs maintain high efficacy in terms of patient satisfaction and the ability to produce satisfactory erections, but urologists must carefully consider the decision to proceed with IPP implantation to minimize postoperative complications [8, 9, 18–21]. Using our multiethnic, urban patient population of 530 individuals undergoing primary IPP implantation, we observed an overall reoperation rate of 12.1%, while 5.5% developed postoperative infections. Further analysis indicated that patients with Peyronie’s disease, hemoglobin A1c levels over 8, active smokers, or EBL ≥ 25cc during the operation were more likely to undergo reoperation for any reason and face a higher risk of infection. Additionally, hypertension and the utilization of an infrapubic approach were identified as additional risk factors for reoperation due to infection.

Our overall reoperation rate of 12.1% aligns with or slightly exceeds previous findings, with a median time to reoperation of 4 months, which is also in-line with or slightly earlier than prior studies [17, 22]. This aligns with the known trend of higher reoperation rates among African-American and Hispanic men in our diverse patient cohort [17]. We found a strong correlation between this overall reoperation rate and active smoking, unsurprising given smokers’ heightened risk of postoperative complications, including infection of all types [23, 24]. We also investigated the impact of Arista™ powder, a hemostatic agent with demonstrated efficacy and utility, on reoperation rates [25–27]. We observed that the use of Arista™ was associated with a decreased odds of reoperation, while EBL ≥ 25cc was associated with an increased odds of reoperation. However, these associations did not persist when specifically examining infection cases, suggesting that reoperation in these cases may instead be linked to issues such as device malfunction or wound dehiscence which prompt later reoperation. Patients with surgical drain insertion had higher reoperation rates, but this result was not significant, and recent literature indicates that drains help prevent hematoma and infection [28]. Notably, patients with Peyronie’s disease had higher overall reoperation rates, even after adjusting for confounders. This contrasts with findings from a study affirming the mechanical reliability of IPPs in Peyronie’s patients, as well as Segal et al., who found no increased rates of postoperative infection [29, 30]. Further research is desired to clarify this discrepancy.

Our reoperation rates specifically due to infection slightly exceed those reported in the literature [31, 32]. Numerous strategies to reduce infection rates have been identified, including the use of infection retardant coatings, a preoperative checklist, and implementing the “No-Touch” technique [12, 13, 33–35]. Additionally, Henry et al. found that a revision washout protocol during reoperation surgery may significantly reduce infection rates [36]. High levels of comorbidities in our cohort may contribute to our infection rates. We observed that patients with hemoglobin A1c levels over 8 had a significantly heightened infection risk. While this finding is consistent with Lipsky et al., a multi-center study by Osman et al. found that A1c levels are not predictive of postoperative infections [37, 38]. Additionally, our association was observed at a lower cutoff compared to prior studies, highlighting the importance of hemoglobin A1c screening and management of diabetes patients prior to surgery [39, 40]. Our findings of increased reoperation due to infection in smokers and hypertensive patients align with those of Lacy et al. [41]. However, conflicting evidence exists regarding the relationship between hypertension and reoperation risk, warranting additional investigation [42].

An interesting finding in our study is the association of higher reoperation rates due to infection when using the infrapubic approach compared to the penoscrotal approach. The literature presents conflicting views on the superiority of these methods in terms of efficacy and safety. Some studies suggest that the infrapubic approach offers shorter operative times and faster return to sexual activity, while others advocate for the penoscrotal approach due to its ability to avoid dorsal nerve injury and superior corporal exposure [43, 44]. Despite this debate, multiple studies directly comparing the two approaches have found no difference in infection rates, which is in contrast with our findings [19, 45, 46]. However, our analysis of infection rates based on surgical approach relies on a small sample size, so these results should be interpreted with caution.

In the present study, implant survival was defined as the continued presence of the primary implant without reoperation or removal surgery, documented at specified time intervals during in-person follow-up. If patients missed their follow-up appointments, their implants were not considered survived. This led to deflated survival rates, particularly at later time points, due to frequent loss to follow-up in our underserved, urban, high-volume academic center. Although our 5-year reoperation survival rate is lower than previous studies due to this limitation, our 1-year survival rates relatively align with those reported in a recent systematic review and meta-analysis [47]. Additional research suggests overall reoperation survival rates with IPP implants may reach as high as 60% after 20 years with satisfactory QoL outcomes [48].

Our study has several additional limitations. The retrospective design poses challenges in data collection, as some information may be missing or inadequately reported during chart review, and it predisposes the study to selection bias. A prospective approach could allow for better communication with patients who might otherwise be lost to follow-up, potentially minimizing bias and improving sample sizes. Small sample sizes are observed at later follow-ups, which increase the risk of bias and make it challenging to draw robust conclusions. Additionally, it is impractical to control for all risk factors predisposing patients to reoperation, and several confounders that were not analyzed in this study likely exist. A single center, single surgeon design limits the study’s generalizability, particularly given our urban, multiethnic patient population. Furthermore, differences in the sample size between groups were considerable, thereby decreasing the statistical power of the study.

In conclusion, our study indicates that patients with Peyronie’s disease, hemoglobin A1c levels over 8, intraoperative EBL ≥ 25cc, and active smoking face higher odds of reoperation for any reason, while the use of Arista™ powder reduces these odds. Specifically for infection-related reoperations, hypertension and the infrapubic approach were identified as additional risk factors. Our findings highlight the importance of counseling patients with identified risk factors prior to surgery about the potential for reoperation and infection. More research is desired to understand the impact of surgical approaches, Peyronie’s disease, and hypertension on reoperation rates. With a better understanding of these drivers of reoperation, we can work to identify and implement strategies to mitigate the risk of reoperation in men undergoing primary IPP surgery.

## Data Availability

The data that support the findings of this study are available on request from the corresponding author. The data are not publicly available due to their containing information that could compromise the privacy of research participants.
